# Predictors of unplanned emergency hospital admissions among patients aged 65+ with multimorbidity and depression in Northwest London during and after the Covid-19 lockdown in England

**DOI:** 10.1371/journal.pone.0294639

**Published:** 2024-02-23

**Authors:** Meryem Cicek, Geva Greenfield, Dasha Nicholls, Azeem Majeed, Benedict Hayhoe

**Affiliations:** 1 Applied Research Collaboration Northwest London (ARC NWL), Department of Primary Care and Public Health, School of Public Health, Faculty of Medicine, Imperial College London, London, United Kingdom; 2 Division of Psychiatry, Department of Brain Sciences, Faculty of Medicine, Imperial College London, London, United Kingdom; University of Oxford, UNITED KINGDOM

## Abstract

**Introduction:**

Individuals with multimorbidity have an increased likelihood of using unplanned secondary care including emergency department visits and emergency hospitalisations. Those with mental health comorbidities are affected to a greater extent. The Covid-19 pandemic has negatively impacted on psychosocial wellbeing and multimorbidity care, especially among vulnerable older individuals.

**Aim:**

To examine the risk of unplanned hospital admissions among patients aged 65+ with multimorbidity and depression in Northwest London, England, during- and post-Covid-19 lockdown.

**Methods:**

Retrospective cross-sectional data analysis with the Discover-NOW database for Northwest London was conducted. The overall sample consisted of 20,165 registered patients aged 65+ with depression. Two time periods were compared to observe the impact of the Covid-19 lockdown on emergency hospital admissions between 23rd March 2020 to 21st June 2021 (period 1) and equivalent-length post-lockdown period from 22^nd^ June 2021 to 19th September 2022 (period 2). Multivariate logistic regression was conducted on having at least one emergency hospital admission in each period against sociodemographic and multimorbidity-related characteristics.

**Results:**

The odds of having an emergency hospitalisation were greater in men than women (OR = 1.19 (lockdown); OR = 1.29 (post-lockdown)), and significantly increased with age, higher deprivation, and greater number of comorbidities in both periods across the majority of categories. There was an inconclusive pattern with ethnicity; with a statistically significant protective effect among Asian (OR = 0.66) and Black ethnicities (OR = 0.67) compared to White patients during post-lockdown period only.

**Conclusion:**

The likelihood of unplanned hospitalisation was higher in men than women, and significantly increased with age, higher deprivation, and comorbidities. Despite modest increases in magnitude of risk between lockdown and post-lockdown periods, there is evidence to support proactive case-review by multi-disciplinary teams to avoid unplanned admissions, particularly men with multimorbidity and comorbid depression, patients with higher number of comorbidities and greater deprivation. Further work is needed to determine admission reasons, multimorbidity patterns, and other clinical and lifestyle predictors.

## Introduction

There is an increasing prevalence of multimorbidity, the co-occurrence of two or more long-term conditions, among populations globally [1] including the UK [2–4] comprising 27.2% in people aged 18 and over [3]. The number of People with 4 or more conditions is predicted to increase, particularly among 65+ year-olds, from 9.8% in 2015 to 17% in 2035 [5]. For many of these individuals, one or more conditions will be a mental health comorbidity [3, 4], either common- or severe mental illness, or a combination of both. Mental illnesses such as depression or anxiety are common in the general population and as comorbidities among multimorbid patients. Incident depression increases by 45% with every additional physical condition an individual has [6], making it a leading cause of disability globally [7] and in the UK [8].

The presence of a common mental health comorbidity like depression among patients with physical long-term conditions increases an individual’s healthcare utilisation [3, 9, 10], including unplanned secondary care [11–15]. This includes visits to emergency departments, unplanned hospital admissions and unplanned readmission within 30 days of discharge from hospital. A few previous studies have looked at the effect of comorbid depression on unplanned hospital care in Sheffield [13], and Yorkshire in England [14], and Scotland [12]. However, no further studies to date have explored the predictors of unplanned hospitalisations in other areas in the UK, including London, and have not been recently conducted since the Covid-19 pandemic occurred.

Since the onset of the Covid-19 pandemic and following lockdown in early 2020, there has been a reported increase in depression incidence in the UK [16, 17]. However, the reliability of these incidence figures may be affected by under-reporting related to delays in access to healthcare services due to the pandemic and annual winter period pressures [18]. Recent evidence globally has reported increases in depressive symptomology in patients with multimorbidity and the negative impact of the pandemic on missed appointments and deterioration in psychosocial wellbeing [18–20]. The prevalence of depression among those with multimorbidity poses a risk of potential unmet need or exacerbation of the condition, particularly among vulnerable older patients experiencing loneliness, physical impairment such as pain, and difficulties with accessing health services throughout the pandemic [18].

In a healthcare system under significant pressure, especially emerging from the pandemic, identifying and addressing the risk of unplanned healthcare use is of critical importance. Amongst individuals with multimorbidity including mental health conditions, who are already at higher risk of morbidity, the identification of specific predictors of unplanned healthcare use will be key to improving both patient health outcomes and experience of healthcare. To our knowledge, there is no study exploring this association in two cohorts, during and after the Covid-19 pandemic lockdown. Thus, the aim of this paper is to understand the predictors of unplanned hospital admissions among patients with multimorbidity and depression, among those aged 65+ in Northwest London. The study seeks to a) describe and investigate the predictive nature of the sociodemographic characteristics of 65+ year-olds with multimorbidity and depression in Northwest London, and b) observe the impact of the Covid-19 pandemic on emergency hospital admissions in this sub-population comparing lockdown and post-lockdown periods.

## Methods

### Data sources

For this retrospective cross-sectional study, the Discover-NOW database, housing the Whole Systems Integrated Care (WSIC) dataset, was used. This is an integrated dataset of patients across Northwest London (NWL) which allows for linking of deidentified patient data across routine National Health System (NHS) primary care, secondary care, mental health, social and community care [21]. The integrated Secondary Uses Service (SUS) data provided information on non-elective (i.e., emergency) hospital admission spells, linked with General Practice (GP) primary care data with information on sociodemographic predictors and diagnosis of comorbidities. There is no access to any identifiable patient information during or after data collection, as the Discover-NOW dataset is fully deidentified.

### Study time periods

Two equivalent-length periods were compared: the full Covid-19 lockdown period in England, encompassing the first lockdown to the end of the last lockdown (Period 1: during Covid-19 lockdown, 23rd March 2020 to 21st June 2021–14 months, 4 weeks); and the period post-lockdown with an equivalent-length (Period 2: post-lockdown, 22nd June 2021 to 19th September 2022–14 months, 4 weeks). The rationale for two period cohorts was to control for the potential confounding due to precarious circumstances and strain on the healthcare system during the pandemic lockdown, and changes in service provision and access during lockdown. Thus, deidentified data from 23^rd^ March 2020 to 19^th^ September 2022 was used.

### Sample population

The sample population included individuals aged 65 and over who are registered with a GP in Northwest London, with active depression during both study periods 1 and 2. The inclusion criteria for participants was to capture individuals those with depression, with and without comorbidity. Depression was defined by having at least one active depression Systematized Nomenclature of Medicine Clinical Terms (SNOMED CT) code in their GP primary care records within each of the respective study periods. The full list of the SNOMED CT codes used were independently reviewed by two GP clinicians for suitability to reduce the potential misclassification of depression (see S1 Table). The data was cleaned to exclude any records with missing data for the key sociodemographic predictors investigated i.e., sex, age, deprivation and ethnicity, to arrive at overall study size of 20,165 across both time periods.

### Predictor variables

Multimorbidity was defined as two or more conditions, i.e., at least one other condition besides depression in the defined sample population. For the long-term conditions included in our multimorbidity definition, we used the latest version of the UK Quality Outcomes Framework (QOF) list of conditions from 2021/2022 guidelines [22] and their SNOMED CT diagnostic codes used in the Primary Care Domain Reference Set Portal available from NHS Digital [23]. The sociodemographic variables included were sex, age, deprivation (using Index of Multiple Deprivation (IMD) decile), and ethnicity.

### Outcome

An unplanned hospital admission is defined in the NHS England SUS data as either an ‘episode’, a single consultation or a ‘spell’ which is a continuous period of time spent as a patient within an NHS trust, that may involve more than one episode [24]. Unplanned admission ‘spells’ were chosen over ‘episodes’ because there may be multiple episodes in a single visit to the same secondary care provider. This mitigates exaggerating varying episodes which may involve short procedures or tests grouped under the spell, and thus reduces potential misclassification of admissions. The observations for unplanned admission spells were treated as a binary outcome (Yes = 1 or No = 0) for the individual having at least one unplanned admission in the given time period.

### Data analysis

A descriptive analysis on the sociodemographic and multimorbidity characteristics of the sample population was conducted by examining age categories, sex, deprivation (using IMD Decile), ethnic category, and number of comorbidities in the study population of those with depression. This was conducted for both periods to determine whether the distribution of these characteristics varied across study periods. Multivariate logistic regression models were conducted to predict the binary outcome (having at least one emergency hospital admission spell) based on the predictors, as categorical variables. Data was analysed retrospectively in October 2022. The study was conducted in accordance with the ‘Strengthening the Reporting of Observational Studies in Epidemiology (STROBE)’ guidelines (see S2 Table).

### Ethical approval

Ethical approval was granted by the Northwest London CCG Sub-Committee for WSIC to use de-identified patient data. This approval is bound by the Health Research Authority (HRA) ethics agreement which is in place for WSIC until 2023. There was no identifiable confidential patient data that was available nor used in this research.

## Results

### Study population

Overall, a total of 20,165 adults with an active depression clinical code registered in Northwest London across both periods (period 1 = 10,361, period 2 = 9,804), were included in the study sample after excluding ineligible records with missing sociodemographic data. There was a slightly larger proportion of women in both period cohorts, across those with and without an admission (Table 1). The age distribution in both periods was similar with 65–74-year-olds comprising the largest age group, reflecting normal population distribution. There was a larger proportion of individuals belonging to White ethnic group, approximately distributed at 60% across both periods and sub-groups of those with and without an admission. Most of the sample population belonged IMD Decile categories between 3–6 where 1 was the most deprived and 10 the least deprived. Approximately only 7% of the sample had only depression, without multimorbidity, while the majority of the population (61% in period 1; 59% in period 2) had 1–3 comorbidities prevalent (Table 1).

**Table 1:** Characteristics of the adult population aged 65+with multimorbidity and active depression in Northwest London during Covid-19 lockdown (Period 1, 23^rd^ Mar 2020 – 21^st^ June 2021) and post Covid-19 lockdown (Period 2, 22^nd^ June 2021 – 19^th^ Sept 2022).

Characteristic	Period 1			Period 2		
	Overall, N = 10,361 (%)	Did not have an admission, N = 7,258 (%)	Had an admission, N = 3,103 (%)	Overall, N = 9,8041 (%)	Did not have an admission, N = 6,739 (%)	Had an admission, N = 3,065 (%)
**Sex**						
Female	6,104 (59%)	4,302 (59%)	1,802 (58%)	5,953 (61%)	4,194 (62%)	1,759 (57%)
Male	4,257 (41%)	2,956 (41%)	1,301 (42%)	3,851 (39%)	2,545 (38%)	1,306 (43%)
**Age**						
65–74	5,042 (49%)	4,069 (56%)	973 (31%)	4,644 (47%)	3,673 (55%)	971 (32%)
75–84	3,265 (32%)	2,174 (30%)	1,091 (35%)	3,208 (33%)	2,085 (31%)	1,123 (37%)
85–94	1,786 (17%)	898 (12%)	888 (29%)	1,726 (18%)	876 (13%)	850 (28%)
95+	268 (2.6%)	117 (1.6%)	151 (4.9%)	226 (2.3%)	105 (1.6%)	121 (3.9%)
**Ethnicity**						
White	6,082 (59%)	4,058 (56%)	2,024 (65%)	5,778 (59%)	3,766 (56%)	2,012 (66%)
Asian or Asian British	2,660 (26%)	2,088 (29%)	572 (18%)	2,409 (25%)	1,845 (27%)	564 (18%)
Black or Black British	741 (7.2%)	478 (6.6%)	263 (8.5%)	718 (7.3%)	489 (7.3%)	229 (7.5%)
Mixed	198 (1.9%)	143 (2.0%)	55 (1.8%)	205 (2.1%)	146 (2.2%)	59 (1.9%)
Other ethnic groups	649 (6.3%)	461 (6.4%)	188 (6.1%)	670 (6.9%)	470 (7.0%)	200 (6.5%)
**IMD Decile **						
1 (most deprived)	616 (5.9%)	366 (5.0%)	250 (8.1%)	685 (7.0%)	412 (6.1%)	273 (8.9%)
2	1,064 (10%)	651 (9.0%)	413 (13%)	1,077 (11%)	660 (9.8%)	417 (14%)
3	1,558 (15%)	1,037 (14%)	521 (17%)	1,511 (15%)	989 (15%)	522 (17%)
4	1,413 (14%)	992 (14%)	421 (14%)	1,311 (13%)	884 (13%)	427 (14%)
5	1,259 (12%)	889 (12%)	370 (12%)	1,175 (12%)	822 (12%)	353 (12%)
6	1,224 (12%)	874 (12%)	350 (11%)	1,237 (13%)	870 (13%)	367 (12%)
7	1,063 (10%)	755 (10%)	308 (9.9%)	904 (9.2%)	652 (9.7%)	252 (8.2%)
8	700 (6.8%)	529 (7.3%)	171 (5.5%)	683 (7.0%)	491 (7.3%)	192 (6.3%)
9	673 (6.5%)	529 (7.3%)	144 (4.6%)	598 (6.1%)	444 (6.6%)	154 (5.0%)
10 (least deprived)	791 (7.6%)	636 (8.8%)	155 (5.0%)	623 (6.4%)	515 (7.6%)	108 (3.5%)
**Number of Comorbidities**						
0	712 (6.9%)	584 (8.0%)	128 (4.1%)	743 (7.6%)	581 (8.6%)	162 (5.3%)
1–3	6,288 (61%)	4,534 (62%)	1,754 (57%)	5,737 (59%)	4,213 (63%)	1,524 (50%)
4–6	2,876 (28%)	1,880 (26%)	996 (32%)	2,826 (29%)	1,714 (25%)	1,112 (36%)
7–9	464 (4.5%)	252 (3.5%)	212 (6.8%)	474 (4.8%)	220 (3.3%)	254 (8.3%)
10+	21 (0.2%)	8 (0.1%)	13 (0.4%)	24 (0.2%)	11 (0.2%)	13 (0.4%)

### Unplanned emergency hospital admissions during Covid-19 lockdown and post-lockdown periods

The overall pattern of the risk of unplanned emergency hospital admission between period 1 and period 2, was similar in the direction and magnitude of association across all predictors (Table 2). The difference in the odds ratios (ORs) between the categories of predictors were similar across both periods (Table 2).

**Table 2:** Multivariate logistic regression model of risk of at least one emergency hospital admission during the Covid-19 lockdown (period 1) and post-lockdown (period 2).

Characteristic	Period 1: Lockdown (n = 10,361)			Period 2: Post-Lockdown (n = 9,804)		
	OR	95% CI	p-value	OR	95% CI	p-value
**Age**						
65–74 (ref)	—	—		—	—	
75–84	2.03	1.83, 2.25	<0.001	1.94	1.75, 2.15	<0.001
85–94	3.79	3.36, 4.28	<0.001	3.29	2.91, 3.73	<0.001
95+	5.00	3.87, 6.48	<0.001	4.04	3.06, 5.35	<0.001
**Sex**						
Female (ref)	—	—		—	—	
Male	1.19	1.09, 1.31	<0.001	1.29	1.17, 1.41	<0.001
**Deprivation (IMD Decile)**						
1 (most deprived)(ref)	—	—		—	—	
2	0.98	0.79, 1.22	0.9	1.04	0.85, 1.28	0.7
3	0.82	0.67, 1.01	0.056	0.85	0.70, 1.04	0.11
4	0.70	0.57, 0.86	<0.001	0.78	0.64, 0.95	0.015
5	0.70	0.57, 0.87	0.001	0.73	0.60, 0.91	0.004
6	0.63	0.50, 0.78	<0.001	0.65	0.53, 0.80	<0.001
7	0.62	0.49, 0.77	<0.001	0.59	0.47, 0.74	<0.001
8	0.50	0.39, 0.64	<0.001	0.58	0.46, 0.74	<0.001
9	0.40	0.31, 0.52	<0.001	0.52	0.41, 0.67	<0.001
10 (least deprived)	0.37	0.29, 0.47	<0.001	0.32	0.24, 0.42	<0.001
**Ethnicity**						
White (ref)	—	—		—	—	
Asian or Asian British	0.67	0.59, 0.75	<0.001	0.66	0.59, 0.74	<0.001
Black or Black British	0.87	0.73, 1.04	0.12	0.67	0.56, 0.80	<0.001
Mixed	0.74	0.53, 1.03	0.077	0.75	0.54, 1.02	0.074
Other ethnic groups	0.94	0.78, 1.14	0.5	0.82	0.68, 0.98	0.032
**Number of Comorbidities**						
0 (ref)	—	—		—	—	
1–3	1.56	1.27, 1.93	<0.001	1.15	0.95, 1.39	0.2
4–6	1.94	1.57, 2.41	<0.001	1.85	1.52, 2.26	<0.001
7–9	2.65	2.01, 3.50	<0.001	2.83	2.18, 3.69	<0.001
10+	4.48	1.80, 11.8	0.002	3.03	1.29, 7.26	0.011

OR = Odds Ratio, CI = Confidence Interval

The greater the number of comorbidities an individual has, the greater the risk of having at least one unplanned emergency hospital admission during both periods. When compared to individuals without multimorbidity (i.e., 0 comorbidities, only depression), those with 1–3 comorbidities had a 1.56 times greater risk of emergency hospitalisation during the lockdown period, to 1.15 times greater risk in the post-lockdown period, although this was not statistically significant for the post-lockdown period. However, when observing all other comorbidity categories, there is a statistically significant positive gradient as the number of comorbidities increases beyond four comorbidities, which is true for both periods studied. For example, when compared to a reference group of individuals without any comorbidities, individuals with 4–6 conditions had a 1.94 times higher risk of emergency hospitalisation during lockdown compared to 1.85 greater risk post-lockdown. This significantly increases when the number of comorbidities increases to 7–9 (2.65 vs. 2.83) and further with more than 10 comorbidities (4.48 vs 3.03), respectively.

The odds of having at least one unplanned emergency hospital admission during the Covid-19 lockdown (period 1) was 1.19 times greater for men than women, while this increased to 1.29 times greater for men in the post-lockdown period (period 2). There is a statistically significant gradient of positive association by age bands, whereby compared to those aged 65–74, the odds of an emergency admission were double for those aged 75–84 in both periods. This increases to 3.79 times and 3.29 times greater for those aged 85–94 in periods 1 and 2, respectively.

Greater deprivation was associated with increased likelihood of having an emergency admission in both periods. There is a gradient of positive association by stratified deprivation, especially statistically significant through IMD deciles 4 to 10 across both study periods. In period 1, IMD decile 4 had 0.70 times the risk of an unplanned admission compared to 0.37 times the risk for people in IMD decile 10, when compared to the reference category IMD 1, the most deprived group.

## Discussion

### Principal findings

The likelihood of having at least one unplanned emergency hospital admission in adults aged 65+ with active depression and multimorbidity did not significantly differ when comparing the two periods of Covid-19 lockdown (period 1) and post-lockdown (period 2). Men had a higher risk of having an emergency hospital admission, compared to women, and this difference was greater post-lockdown compared with during lockdown. Older age groups had an incrementally higher risk of admission, as expected in both periods. Greater deprivation was associated with increased likelihood of having an emergency admission in both periods. There was an incremental increase in the risk of admission with the number of comorbidities; the higher the number of comorbidities an individual had, the greater the risk of having at least one unplanned emergency hospital admission during both periods. When compared to individuals with only depression, those with 4–6 additional comorbidities had a 2.65 times greater risk of emergency hospitalisation during the lockdown period, to 2.83 times greater risk in the post-lockdown period. While the risk was not statistically significant for those with 1–3 comorbidities in the post-lockdown period, all other categories of increasing comorbidity showed a strong positive gradient that was statistically significant. However, the results with ethnicity were less conclusive, with Asian and Black ethnicity providing a modest protective effect when compared to White ethnicity during the post-lockdown period.

### Contextualising findings

The primary aim of this study was to compare findings on risk of emergency hospitalisation among individuals with depression and multimorbidity from the two different time periods related to Covid-19 lockdown and post-lockdown in Northwest London. The results indicate that during the Covid-19 lockdown period, the risk of emergency hospital admission was slightly greater across all age groups and categories of the number of comorbidities an individual had compared to the post-lockdown period. This may be an indication of emergency hospitalisations due to Covid or exacerbated existing multimorbidity during this lockdown period perhaps due to less access to primary care and impacting on receiving adequate care of long-term conditions. However, the magnitude of association was only slightly greater, on the whole, in the lockdown period compared to post-lockdown, which may be due to potential spill-over effects of the lockdown on service access into the post-lockdown period.

We found that individuals with depression alongside 1–3 comorbidities were more likely to have a hospital admission than those without comorbidities (i.e., only depression). The risk of unplanned admission consistently increased with higher numbers of comorbidities (a measure of greater severity of multimorbidity) in our study, as established in the literature [11–15]. These findings are aligned with a survey demonstrating that depression increased the risk of prospective emergency hospital admissions by 1.58 times among primary care patients with other long-term conditions [12].

Considering the positive association between deprivation and risk of unplanned admissions to hospital, our findings are concordant with a large Scottish study that showed a clear gradient of positive association between unplanned hospitalisations and categories of deprivation & the number of physical conditions [11]. Although the study population age ranges are different from our study (adults aged 20+ versus aged 65+), the gradient of positive association between deprivation and unplanned hospitalisations is apparent [11], and is also reflected in other larger studies examining healthcare utilisation among patients with multimorbidity [2, 4]. Interestingly, when surveyed by the ELSA Covid-19 Substudy, people with multimorbidity aged 65+ in the early pandemic found that 14% of people did not attempt accessing social and community health services during this period, while 20% who did want access to these services did not have access [25]. This may point to sociodemographic inequalities to accessing certain services that may have otherwise helped with community-based management of multimorbidity. Furthermore, it may have potentially led to either using emergency care inappropriately or lack of adequate access to community-based healthcare services which could have cause latent effects in the post-lockdown period.

### Strengths and limitations

#### Strengths

A key strength is that equivalent-length time periods were used to compare the effects of the Covid-19 lockdown, and post-lockdown period, to address any confounding arising from differences in reduced accessibility to services or limited health service provision in emergency secondary care. Also, an integrated database (DiscoverNow WSIC) was used, which allowed for linking up of patient characteristics across clinical data sources from primary care and secondary care, which are typically unavailable with other data infrastructure. Furthermore, this was a timely analysis given the current context of having emerged from Covid-19 lockdown for a substantial period, which can provide some insight into the after-effects of the pandemic on emergency health service utilisation. Moreover, given the particularly adverse impact of the Covid-19 pandemic and lockdowns on people aged 65+, on their mental and physical wellbeing, this study provides some insight any health inequalities between sociodemographic groups and understand which people with multimorbidity might be further investigated.

#### Limitations

While this study has some strengths, there are also some limitations. Firstly, considering the older age band, fluctuations in the sample population due to death from Covid or other causes may have led to some differences in the distribution of population characteristics, although this appears to be minimal. The temporal changes in the multimorbidity status of individuals across a range of conditions may have been difficult to capture precisely over the period studied due to data input limitations. This is similar with understanding the duration of depression diagnosis as it may be episodic or recurrent. We were not able to identify what proportion of patients were in both samples, so this could account for similar findings at both time points. Also, the predictors focused on in this analysis were sociodemographic predictors to understand if there were any health inequalities in the population. There were not as many clinical characteristics beyond the number of comorbidities the sub-population of those with active depression had. Finally, there is a lack of information on the reason for the unplanned emergency hospital admission; this limits our insight on whether admission was Covid-19 related, if there was a pattern in the adverse event leading to admission, or lack of accessibility of other services. This would warrant further investigation with a ‘normal’ period pre-pandemic, or a future post-pandemic period.

### Implications for policy and practice

This study provides some insights on unplanned secondary care in patients with multimorbidity, which is a key focus of the national shift to integrated care strategies in the NHS. The identification of individuals within the group of depression and multimorbidity who are at higher risk of unplanned admissions is important for public health action try to reduce their likelihood of admission. Although the findings were not particularly unexpected across the patient characteristics, and minimal differences were observed between the two lockdown and post-lockdown periods, these findings support existing evidence on the increased risk of admission for higher deprivation and greater number of comorbidities. While the difference between men and women was modest but statistically significant across both periods, this may warrant understanding the health-seeking behaviours for unplanned care in those men with comorbid depression potentially. Considering these findings, multidisciplinary teams in primary care may be encouraged to dedicate proactive case review of men with depression and multimorbidity, through utilising electronic health record filters. Similarly, hospitals could highlight those admitted patients to feedback to GP practices for proactive review, flagging them as individuals at high risk of re-admission to hospital.

### Future research

This study provides some insights into the likelihood of having at least one unplanned emergency hospital admission among those with multimorbidity and depression, among people aged 65+. However, there is scope for future work to build on these findings. While the 65+ group was initially prioritised in this analysis, due to wider implications of having negative impact of Covid-19 pandemic on mental and physical wellbeing, as well as safety and accessibility to healthcare services, this research should be expanded to other age groups to gain a full population perspective. This may identify patterns of emergency service use and reasons for admission in greater granularity. Also, clustering of diseases using k-means may be conducted to understand the particular effects of condition combinations in a larger study population. Additionally, there may be further research on other clinical characteristics or lifestyle habits that may provide additional information on other predictors of unplanned emergency hospitalisations.

There is a need to further investigate the primary reason for admission and primary treatment to understand the differences for reasons for admission. This may offer insight on where there were latent effects of Covid-19 on patients’ multimorbidity condition or frailty, and the patterns of unplanned care use to provide useful information on case finding of frequent users. This may be used to prioritise and plan health services to minimise risk of avoidable emergency admissions. Moreover, the comparison of emergency admissions with measures of GP access in the context of patients aged 65+ may be informative, to assess the overall quality of care for multimorbidity from a holistic perspective.

### Conclusion

We found that when Covid-19 lockdown and post-lockdown periods in Northwest London are compared among 65+ year-olds adults with depression and multimorbidity, there was little difference in the likelihood of an emergency hospitalisation between the two periods. Across both periods, the odds of having an emergency hospitalisation were greater in males than females, and significantly increases with age, higher deprivation, greater number of comorbidities, and decreases slightly for Asian and Black ethnicity compared to White ethnicity. There was a modest difference between the lockdown and post-lockdown periods in magnitude of risk of unplanned hospital admission by patient characteristic. There is a need for further investigation into the reasons for admission, multimorbidity patterns, expansion of this research into other age groups, and clarity on other clinical and lifestyle predictors. However, there is clear evidence to support the need for proactive case-finding and case-review by multi-disciplinary teams in primary care to avoid unplanned admission, particularly men with depression and multimorbidity, patients with greater number of comorbidities and higher deprivation.

## Supporting information

S1 Table Defining depression with Systematized Nomenclature of Medicine Clinical Terms (SNOMED CT). Presence of any of the following clinical codes were used to define active depression code in patients’ clinical records during each respective time period.
(DOCX)

S2 Table Strengthening the Reporting of Observational Studies in Epidemiology (STROBE) statement checklist. (DOCX)
