# Supplementary material for: Predictors of unplanned emergency hospital admissions among patients aged 65+ with multimorbidity and depression in Northwest London during and after the Covid-19 lockdown in England
Source: PLoS One. 2024 Feb 23;19(2):e0294639. doi: 10.1371/journal.pone.0294639 (PMC10890757; doi:10.1371/journal.pone.0294639)
Supplement: S1 Table — Presence of any of the following clinical codes were used to define active depression code in patients’ clinical records during each respective time period. (DOCX) [file pone.0294639.s001.docx]

# Supplementary information

## S1 Table. Defining depression with Systematized Nomenclature of Medicine Clinical Terms (SNOMED CT). Presence of any of the following clinical codes were used to define active depression code in patients' clinical records during each respective time period.

| **SnomedTerm** | **SnomedConceptID** |
| --- | --- |
| Depressive disorder | 35489007 |
| Mixed anxiety and depressive disorder | 231504006 |
| Depression interim review | 413973005 |
| Depressed mood | 366979004 |
| Depression medication review | 413974004 |
| Depression screening using questions | 200971000000100 |
| Depression annual review | 413972000 |
| Recurrent depression | 191616006 |
| Reactive depression (situational) | 87414006 |
| Postnatal depression counselling | 395072006 |
| Moderate depression | 310496002 |
| Mild depression | 310495003 |
| Chronic depression | 192080009 |
| Single major depressive episode, severe, with psychosis | 191604000 |
| Severe depression | 310497006 |
| Major depression, single episode | 36923009 |
| Severe major depression with psychotic features | 73867007 |
| [X]Moderate depressive episode | 465441000000108 |
| Depression monitoring invitation SMS (short message service) text message | 1083071000000100 |
| Mild postnatal depression | 237349002 |
| Postpartum depression | 58703003 |
| Depression monitoring invitation | 711451000000102 |
| Suspected depressive disorder | 473126001 |
| Patient given advice about management of depression | 415044007 |
| Reactive depressive psychosis | 191676002 |
| Recurrent major depressive episodes, severe, with psychosis | 191613003 |
| Moderate major depression | 832007 |
| Antidepressant drug treatment started | 764639002 |
| Agitated depression | 83458005 |
| Recurrent major depressive episodes | 268621008 |
| [X]Severe depressive episode without psychotic symptoms | 397701000000102 |
| Severe recurrent major depression with psychotic features | 28475009 |
| Major depressive disorder | 370143000 |
| Depression monitoring invitation email | 1083051000000100 |
| Chronic depressive personality disorder | 442057004 |
| Recurrent major depressive episodes, moderate | 191611001 |
| Antidepressant drug treatment changed | 823501000000106 |
| Single major depressive episode, severe, with psychosis, psychosis in remission | 755321000000106 |
| Severe major depression without psychotic features | 75084000 |
| Recurrent major depressive episodes, severe, with psychosis, psychosis in remission | 755331000000108 |
| Endogenous depression | 300706003 |
| Depression worse in morning | 247801000 |
| Recurrent major depressive episodes, severe | 764611000000100 |
| Moderate major depression, single episode | 15639000 |
| Severe major depression, single episode | 251000119105 |
| Maternal postnatal depression | 1038261000000100 |
| Mild major depression | 87512008 |
| Endogenous depression - recurrent | 274948002 |
| Severe postnatal depression | 237350002 |
| Severe recurrent major depression without psychotic features | 36474008 |
| Mild recurrent major depression | 40379007 |
| Recurrent brief depressive disorder | 40568001 |
| Follow up for depression | 870191006 |
| Depressive episode | 871840004 |
| Recurrent major depressive episodes, mild | 191610000 |
| Mild major depression, single episode | 79298009 |
| Antenatal depression | 790961000000101 |
| Recurrent major depression in full remission | 46244001 |
| History of postnatal depression | 311611000000104 |
| Prolonged depressive adjustment reaction | 192049004 |
| Signposting to depression self-help group | 1057351000000100 |
| Endogenous depression first episode | 231499006 |
| Atypical depressive disorder | 191659001 |
| Referral for guided self-help for depression | 199111000000100 |
| Brief depressive adjustment reaction | 192046006 |
| Adjustment disorder with depressed mood | 57194009 |
| Depression worse later in day | 247802007 |
| Senile dementia with depression | 191459006 |
| Recurrent reactive depressive episodes, severe, with psychosis | 1086471000000100 |
| Depressive disorder in remission | 698957003 |
| Antidepressant therapy | 698456001 |
| Single episode of major depression in full remission | 19527009 |
| Senile dementia with depressive or paranoid features | 191457008 |
| Major depression single episode, in partial remission | 70747007 |
| Recurrent depression with current severe episode without psychotic features | 1089631000000100 |
| Reactive depression, prolonged single episode | 1086661000000100 |
| Recurrent moderate major depressive disorder co-occurrent with anxiety | 16264901000119100 |
| Positive screening for depression on PHQ-9 (Patient Health Questionnaire 9) | 112001000119100 |
| Recurrent depression with current severe episode and psychotic features | 1089511000000100 |
| Recurrent mild major depressive disorder co-occurrent with anxiety | 16264621000119100 |
| Referral for guided self-help for depression declined | 933441000000101 |
| Depressive personality disorder | 1084061000000100 |
| Counselling for depression | 286711000000107 |
| Post-schizophrenic depression | 231485007 |
| Recurrent depression with current moderate episode | 1089641000000100 |
| Presenile dementia with depression | 191455000 |
| Recurrent major depressive episodes, in partial remission | 764691000000109 |
| Moderately severe major depression single episode | 720453001 |
| Major depressive disorder, single episode with catatonic features | 69392006 |
| Severe major depression, single episode, without psychotic features | 76441001 |
| Masked depression | 231500002 |
| Arteriosclerotic dementia with depression | 191466007 |
| Depressed mood in Alzheimer's disease | 142001000119106 |
| Recurrent severe major depressive disorder co-occurrent with anxiety | 16264821000119100 |
| Postviral depression | 192079006 |
| Recurrent major depression | 66344007 |
| Referral for depression self-help video | 923921000000104 |
| Seasonal affective disorder | 247803002 |
| Dysthymia | 78667006 |
